# Rapid loss of complex polymers and pyrogenic carbon in subsoils under whole-soil warming

**DOI:** 10.1038/s41561-023-01142-1

**Published:** 2023-03-30

**Authors:** Cyrill U. Zosso, Nicholas O. E. Ofiti, Margaret S. Torn, Guido L. B. Wiesenberg, Michael W. I. Schmidt

**Affiliations:** 1 Department of Geography, University of Zurich, Zurich, Switzerland; 2 Climate Sciences Department, Lawrence Berkeley National Laboratory, Berkeley, CA USA; 3 Energy Resources Group, University of California, Berkeley, CA USA

**Keywords:** Carbon cycle, Projection and prediction, Carbon cycle

## Abstract

Subsoils contain more than half of soil organic carbon (SOC) and are expected to experience rapid warming in the coming decades. Yet our understanding of the stability of this vast carbon pool under global warming is uncertain. In particular, the fate of complex molecular structures (polymers) remains debated. Here we show that 4.5 years of whole-soil warming (+4 °C) resulted in less polymeric SOC (sum of specific polymers contributing to SOC) in the warmed subsoil (20–90 cm) relative to control, with no detectable change in topsoil. Warming stimulated the subsoil loss of lignin phenols (−17 ± 0%) derived from woody plant biomass, hydrolysable lipids cutin and suberin, derived from leaf and woody plant biomass (−28 ± 3%), and pyrogenic carbon (−37 ± 8%) produced during incomplete combustion. Given that these compounds have been proposed for long-term carbon sequestration, it is notable that they were rapidly lost in warmed soils. We conclude that complex polymeric carbon in subsoil is vulnerable to decomposition and propose that molecular structure alone may not protect compounds from degradation under future warming.

## Abstract (web summary)

Structurally complex polymeric compounds, such as pyrogenic carbon, that have been previously considered long-term carbon sinks in soils can rapidly be lost by decomposition at warmer temperatures, according to 4.5 years of whole-soil warming experiments.

## Main

Despite evidence that the predominant response of soils to global warming is an increase in heterotrophic respiration^1–3^, it remains uncertain how global warming will affect soil carbon stocks^4^. One source of uncertainty is a lack of process understanding of soil organic carbon (SOC) dynamics in subsoils (here defined as below 20 cm depth; details in Methods). Subsoils store more than half of global SOC^5^, and climate models project that subsoils will warm in near synchrony with air and topsoils^6^. Despite their large stock size, subsoils remain understudied and are largely underrepresented in warming studies.

Carbon dynamics in soils are regulated by multiple biotic and abiotic factors, many of which can differ between topsoil and subsoil^7^. For example, whereas above-ground litter may be an important carbon input to topsoil, roots become more important inputs with increasing soil depth^8^. Depending on biotic and abiotic factors, specific molecular structures might preferentially degrade or accumulate^9^, and some complex molecular structures have been proposed to remain in the soil on centennial timescales^10–13^. A potential climatic control on the molecular composition of SOC is temperature since temperature may affect relative decomposition rates. Simple kinetic theory predicts that decomposition of a complex substrate (higher activation energy) is more temperature sensitive compared with simple substrates^14^, if other biotic and abiotic processes are excluded^15^. A meta-analysis on enzymatic activity in warmed soils indicated that in situ warming stimulates enzymes responsible for the decomposition of complex substrates (ligninase) more than it does enzymes responsible for the decomposition of simple substrates (cellulase)^16^. If this temperature effect dominates the varying influences of biotic and abiotic factors on decomposition under future warming, complex molecular structures that are to date predicted to remain in soils for long periods could be especially vulnerable to decomposition.

One polymeric compound that is considered stable in most soils due to its polyaromatic chemical structure is pyrogenic carbon (PyC), derived from incomplete combustion processes^17^. However, recent findings challenge the millennial persistence of PyC in soils, showing that PyC turns over on a timescale of centuries^13^—with a mean residence time about 1.5 times that of bulk SOC^11^. The wood-derived polymer lignin, which is made up of phenolic structures, has been described and modelled as part of the recalcitrant carbon pool, but it too has been shown to readily degrade in soils^12,18^. Finally, plant-derived hydrolysable lipids cutin, derived from cuticular tissues of vascular plants^19,20^, and suberin, derived from woody tissues^20^, are being considered useful for carbon sequestration^21^ due to their chemical structure^22^ and the assumption that they have longer turnover times than does bulk SOC^10^. It is crucial to better understand how polymeric subsoil SOC is affected by increasing temperatures, more so when such polymeric compounds are being considered for carbon sequestration purposes^17,21^.

In this Article, we made use of one of the first multi-year, whole-soil-profile warming experiments, located at the University of California Blodgett Experimental Forest, to assess warming effects on the composition and degradation of SOC pools at different depths. Specifically, we assessed the fate of plant polymers and PyC after 4.5 years of continuous warming using molecular proxies (lignin, hydrolysable lipids and benzene polycarboxylic acids). The experimental set-up consists of three blocks of paired circular plots (detailed description by ref. ^23^). In warmed plots, vertical heating rods inserted to 2.4 m soil depth provided continuous warming of the soil profile to 1 m depth, in concert with diurnal and seasonal temperature variations^24^. The target warming magnitude was +4 °C based on Representative Concentration Pathway 8.5 predictions for the study region by 2100^25^. This unique set-up allowed us to explore in situ soil carbon responses to warming. Previous work at the study site demonstrated substantial loss of bulk SOC stocks (by 33%) in the subsoil, and soil respiration was consistently increased at all depths over 4.5 years of warming^26^. Through polymer class-specific measurements in this experiment, we show that polymer classes that have been proposed as highly stable^10–13^ are in fact also vulnerable to loss with soil warming such as that expected over this century.

## Large loss of subsoil polymeric SOC but not topsoil

**Fig. 1:**
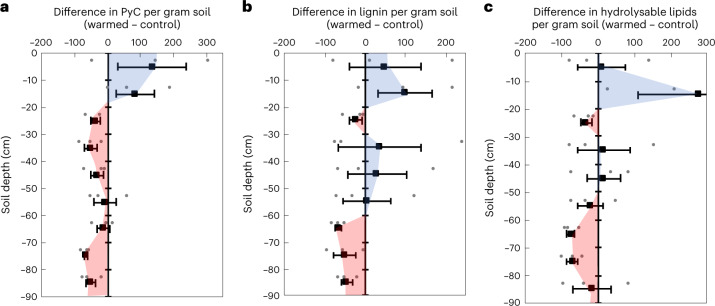
Changes in abundance of PyC and plant polymers at different depths in response to warming. **a**–**c**, Concentrations of PyC (**a**), lignin (**b**) and hydrolysable lipids (**c**) per gram soil were not significantly different in the topsoil (0–20 cm) but were significantly lower in warmed compared with control subsoils (20–90 cm). Values shown are the differences between the warmed and control plots expressed as a percentage ((warmed – control)/control × 100%), with negative values in red and positive values in blue. Black squares show the mean (*n* = 3), and grey circles indicate the single data points. Error bars represent the standard error of the mean (s.e.m.).

We found large differences between topsoil and subsoil for the fate of polymeric SOC under experimental warming. In the topsoil between 0 and 20 cm depth, absolute concentrations of PyC, lignin and hydrolysable lipids (per gram soil) did not differ significantly between warmed and control plots (Fig. 1a–c). By contrast, in subsoil from 20 to 90 cm soil depth, soil warming led to a loss of polymeric carbon, highlighting the vulnerability of subsoil carbon to decomposition under climate change. After 4.5 years of warming, the absolute concentration of PyC (per gram soil) was 37 ± 8% lower in warmed subsoils (Fig. 1a; *P*_numDF1:denDF26_ < 0.01). Similarly, the wood-derived compound lignin was 17 ± 0% lower (Fig. 1b; *P*_1:26_ < 0.01) in warmed compared with control subsoils. The observed losses of PyC and lignin were corroborated by spectroscopic observations from the same site, where aromatic compounds (indicative of PyC and lignin) were less abundant in warmed compared with control plots^27^. A loss of lignin is consistent with the observation that warming increases the activity of lignin-degrading enzymes^16^. Finally, hydrolysable lipids were 28 ± 3% lower (Fig. 1c; *P*_1:24_ = 0.01) in warmed compared with control subsoils. In subsoil, hydrolysable lipids can be composed predominantly of root-derived suberin^8^. Thus, the decreasing root mass with warming at our site^27^ parallels the decrease of hydrolysable lipids.

## No preferential preservation or loss of polymeric SOC

**Fig. 2:**
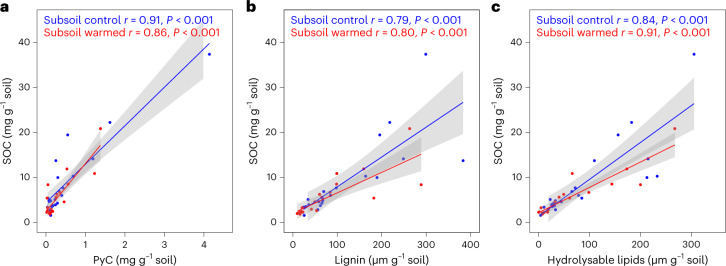
Pearson correlation between bulk SOC and individual polymers shows high correlation of the polymers in control and warmed subsoils. **a**, PyC (control: *r* = 0.91, *P* < 0.001, *n* = 21; warmed: *r* = 0.88, *P* < 0.001, *n* = 21). **b**, Lignin (control: r = 0.79, *P* < 0.001, *n* = 21; warmed: *r* = 0.80, *P* < 0.001, *n* = 21). **c**, Hydrolysable lipids (control: *r* = 0.84, *P* < 0.001, *n* = 21; warmed: *r* = 0.91, *P* < 0.001, *n* = 21). Lines show the two-tailed linear regression of correlation for individual non-transformed data points (*n* = 21), and the grey shaded areas are the confidence intervals (0.95). Regression coefficients and *P* values are given in the respective figures. The blue colours represent information for control subsoil; red represents the warmed subsoil.

Our results show that polymeric SOC was lost at a similar order of magnitude as were bulk SOC stocks (by 33% (ref. ^26^)) due to warming. Concentrations of investigated polymers were strongly correlated with bulk SOC concentrations in both control and warmed subsoils (Fig. 2). Compound carbon concentrations normalized to SOC concentrations did not differ between warmed and control subsoils for PyC (Fig. 2a; *P*_1:26_ = 0.71), lignin (Fig. 2b; *P*_1:26_ = 0.18) or hydrolysable lipids (Fig. 2c; *P*_1:26_ = 0.62). Thus, our results suggest that there was no preferential loss or preservation of any of these polymers.

## Different SOC dynamics in topsoils and subsoils under warming

We report a rapid decrease in concentrations of PyC, lignin and the hydrolysable lipids in subsoils due to in situ experimental warming. These polymeric substances of SOC have been assumed to persist and degrade slower than bulk SOC^10–13^. However, this might vary between ecosystems and soil properties^9^. Importantly, whereas our results show that polymeric C is not preserved preferentially in subsoil with warming, we did not observe a significant effect in topsoil. Thus, the key strength of our method is the uniform warming of the whole profile, which better reflects predictions of whole-soil warming^6^. Nonetheless, experimental warming usually entails limitations due to the experimental set-up, such as effects of temperature magnitude, duration of warming or concomitant drying^28,29^. Some of these limitations will probably also apply for our experimental set-up. For example, at our experimental site, warming caused a slight drying of the topsoil only^26^, which might confound the observed effect of warming.

To our knowledge, only one study has reported changes in polymeric carbon (lignin, suberin and cutin) in warmed subsoil, using infrared heaters^18^. This study on the Tibetan plateau, China, showed an accumulation of suberin and a loss of lignin, potentially due to a shorter period of soil freezing, which allowed for deeper root penetration and enhanced lignin degradation^18^. At our experimental site, root mass was lower in the warmed plots^27^, which could indicate fewer root inputs or faster root turnover. If warming-induced losses of subsoil SOC^26^ are not replaced by, for example, more root growth^18^, then low SOC concentrations in the subsoil might trigger microorganisms to utilize or assimilate complex substrates that might not have been degraded otherwise; alternatively, the stimulation of decomposition enzymes by warming could increase microbial decomposition of all substrates. Likewise, increased fluxes of dissolved organic carbon in warmed plots at our site^26^ might have stimulated the decomposition of polymeric carbon by flushing fresh substrate to the subsoil and alleviating previous resource limitations^30^. Our results highlight that not only simple and labile compounds but also complex polymeric SOC in subsoil might be lost in a warming world due to the higher microbial activity.

Some experiments have investigated the fate of lignin and hydrolysable lipid concentrations under warming in topsoil. Lignin was lost from warmed topsoils in two experiments^31–33^ in contrast to our results of no significant effect. At another experimental site, lignin in topsoil decreased only after more than 20 years of soil warming; this was attributed to changing substrate availability^34^. It remains to be investigated how lignin in topsoil is affected at our site in the long term. The results for hydrolysable lipids are more contrasting. In a hardwood forest, suberin was not affected by 14 months of soil warming, whereas cutin concentrations increased^31^, leading to cutin being considered a ‘heat-proof compound’^35^. In a mixed hardwood forest experiment, concentrations of cutin and suberin significantly decreased after 4 years of soil warming^32^, but these trends were transient and no longer significant after 10 years of warming^33^. Differences in topsoil responses among ecosystems could be related to differences in litter properties. For example, differences in vegetation, among deciduous^31^, mixed hardwood^32,33^ and coniferous forests (our site), result in litter inputs to organic horizons with very different properties and thus degradability. Suseela et al. ^36^ showed that the litter type affects how decomposition responds to warming, which in turn will affect inputs from the litter horizon to topsoil. Besides explaining differences among ecosystems, increasing inputs from the litter horizon to the topsoil at our site could be partially responsible for the difference in how warming affected carbon cycling in the topsoil versus the subsoil.

## Polymeric SOC as an available pool under climate warming

We observed a rapid loss of complex polymers in subsoil due to experimental warming, and our observations indicate that warming stimulated SOC loss at the same rate for PyC, lignin and hydrolysable lipids as for bulk SOC (Fig. 2). The vulnerability to decomposition of SOC in the soil matrix is controlled by a range of biotic and abiotic ecosystem properties^37^ and not only kinetics of individual enzymatic reactions. For example, the physical state of soil organic matter, as free particulate organic matter (POM), occluded POM or mineral-associated organic matter, affects decomposition rates^38,39^, but little is known about temperature sensitivity. At the experimental site, primarily free POM in subsoils was lost, whereas stocks of occluded and mineral-associated carbon did not change with warming^26^. Complex plant-derived compounds might dominantly contribute to POM^12,40^; thus, the fire- and plant-derived polymers lost from these warmed subsoils might have been part of the POM fraction. In the first years of warming, the respired carbon was primarily of young ^14^C age, which aligns well with POM as a primary source^23^. We hypothesize that polymeric SOC will be vulnerable to enhanced decomposition in warmer subsoil because, or if, it is largely found in the unprotected POM.

We highlight the importance of separately considering topsoils and subsoils in predictions of SOC dynamics under warming because the temperature sensitivity of different SOC compounds varies with soil depth. None of the polymeric compounds analysed here proved heat-proof in warmed subsoil, and these compounds decomposed in synchrony with bulk SOC. These findings agree with the paradigm that there is no inherently stable SOC but that the stability depends on ecosystem properties^37^, which also change with soil depth. This study provides crucial knowledge for understanding the long-term fate of compounds that are being considered in carbon sequestration approaches.

## Methods

### Experimental set-up and sampling

The whole-soil warming experiment at the University of California Blodgett Experimental Forest (120°39ʹ40″ W; 38°54ʹ43″ N) was sampled after 4.5 years of whole-soil warming. The experiment was designed to study changes in biogeochemical cycles in a warming climate^23^. The climate is characterized as Mediterranean with mean annual precipitation of 1,660 mm and mean annual air temperature of 12.5 °C (ref. ^26^). The experiment is located on an Alfisol of granitic origin, and the soil is covered by an organic horizon. The vegetation in the coniferous forest is dominated by ponderosa pine (*Pinus ponderosa*), sugar pine (*Pinus lambertiana*), incense cedar (*Calodefrus decurrens*), white fir (*Abies concolor*) and Douglas fir (*Pseudotsuga menziesii*), with understory vegetation consisting of shrubs (mainly *Notholithocarpus densiflorus*), grasses and some other annual plants (for example, *Gallium triflorum*). The experimental set-up has been previously described in detail (ref. ^23^). Briefly, three blocks of paired circular plots (*n* = 3) were set up in October 2013. Plots measure 3 m in diameter, and temperature was elevated in the warmed compared with control plots by 4 °C from 0.2 to 1 m and 2.6 °C above 0.2 m due to surface heat loss. The experimental design preserved diurnal and seasonal temperature variations depth-specifically. Samples were collected in April 2018, after 4.5 years of warming, using a manual soil corer of 4.78 cm diameter. The organic horizons were removed before soil sampling. The mineral soil was sampled in increments of 10 cm to a soil depth of 90 cm. After recovery, the samples were stored cool for transportation and freeze-dried within a week after sampling. Roots were manually recovered from the cores, and the soil was sieved to 2 mm. Ground subsamples were used to measure carbon concentrations on an elemental analyser coupled to an isotope ratio mass spectrometer (EA-IRMS; FLASH 2000 HT Plus, linked by ConFlo IV to DELTA V Plus IRMS; Thermo Fisher Scientific).

**Extended Data Fig. 1:**
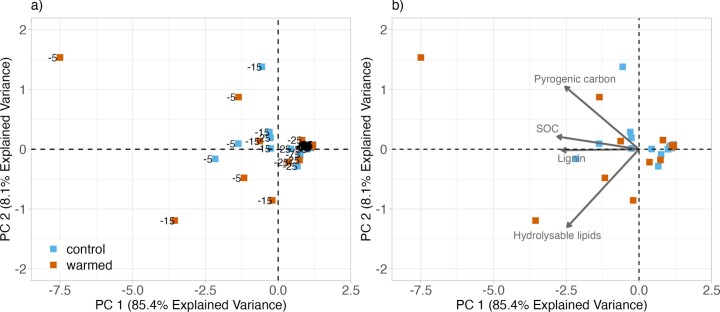
Principal component analysis conducted on SOC, lignin, hydrolysable lipids and pyrogenic carbon. Principal component analysis conducted on SOC, lignin, hydrolysable lipids and pyrogenic carbon, showing a clear separation of the increments from 0–20 cm and 20–90 cm. (**a**) Score plot of the individual soil depth and treatments (n = 54) and (**b**) the corresponding loading plot of the variables (n = 54). PC1 and PC2 explained 85.4% and 8.1% of the variance, respectively.

The differentiation between topsoil and subsoil at a depth of 20 cm is based on the observation by ref. ^26^ that carbon stocks are significantly different between depth increments from 0 to 20 cm and 20 to 90 cm. Furthermore, using principal component analysis with the R function prcomp (54 data points with 4 variables, centred and scaled), depth increments from 0 to 20 cm were clearly separated from the ones from 20 to 90 cm (Extended data Fig. 1).

### Biomarker analysis

#### Pyrogenic carbon

Benzene polycarboxylic acids were used as an approximation of PyC, and the analysis was conducted following the protocol by Wiedemeier et al. (2016) (ref. ^41^) with some modifications. The dried and milled sample containing approximately 10 mg OC was weighed in digestion tubes and digested with nitric acid (65%) at 170 °C for 8 h. A glass fibre filter was used for filtration of the resulting solution. In a next step, the sample was passed over a cation exchange resin for further cleaning and subsequently freeze-dried. The sample was then redissolved in MeOH/H_2_O (1/1), passed over a solid-phase extraction cartridge (C_18_ SPE tube, Supelco), dried again and dissolved in deionized water for the transfer to a high-performance liquid chromatography vial. Separation and quantification were conducted on an Agilent 1290 Infinity high-performance liquid chromatography system (Santa Clara) equipped with an Agilent InfinityLab Poroshell 120 SB-C_18_ column (100 mm × 4.6 mm × 2.7 mm) and coupled to a photo diode array detector. Ortho-phosphoric acid (Honeywell) dissolved in water (pH 1.2–1.3) was used as mobile phase A, and pure acetonitrile (Scharlau) was used as mobile phase B. For quantification, all acids with the different numbers of carboxyl functions were summed (B3CA, B4CA, B5CA and B6CA, respectively).

#### Lignin

Lignin was analysed by alkaline CuO oxidation using microwave digestion to break down the lignin structure into monomers as described in refs. ^42,43^ with some modifications. Briefly, all dried and milled soil samples were analysed in duplicate, and an equivalent of 2–5 mg total organic carbon was weighed into the microwave tubes. After adding 500 mg CuO powder, 50 mg ferrous ammonium sulfate and 20 ml of 2 M NaOH solution, samples were oxidized in the microwave at 150 °C for 90 min. Once cooled, 500 μl of a mixture containing cinnamic acid and ethylvanillin in NaOH (50 mg l^−1^ each) was added as internal standard to each sample, and subsequently the pH was adjusted to pH 2.10 using HCl. Samples were then collected on preconditioned C_18_ columns (DISCOVERY DSC-18 SPE tube, 500 mg; Sigma-Aldrich), eluted 5 times with 500 μl ethyl acetate, dried under N_2_ and redissolved in 400 μl p-anisic acid in ethyl acetate (50 mg l^–1^). Derivatization was done by mixing 70 μl sample with 70 μl N,O-bis (trimethylsilyl) trifluoroacetamide/tetramethylchlorosilane (BSTFA + TCMS; 99/1; Sigma-Aldrich) and heating for 20 min at 60 °C. Compounds were then quantified on an Agilent 7890B gas chromatograph (GC) equipped with a 50 m J&W DB-5ms GC column, a multi-mode injector and a flame ionization detector. Compound identification was supported by external standard and sample measurements on an Agilent 6890 N GC equipped with a split/splitless injector coupled to an Agilent 5973 mass selective detector. Both instruments were equipped with DB-5MS column (50 m × 0.2 mm × 0.33 μm) and 1.5 m de-activated pre-column, with helium as the carrier gas (1 ml min^−1^). The temperature programme was starting at 80 °C and kept isothermal for 5 min, increasing to 110 °C at a rate of 2 °C min^−1^, increasing further to 170 °C at a rate of 0.5 °C min^−1^ and at a rate of 15 °C min^−1^ to 320 °C, where temperature was held again for 10 min. The total concentration of lignin was calculated by summing eight measured oxidation products (vanillin, acetovanillone, vanillic acid, syringaldehyde, acetosyringone, syringic acid, p-coumaric acid and ferulic acid).

#### Suberin and cutin

Cutin and suberin biomarkers were isolated by base hydrolysis to release bound lipids after removal of free lipids, as described in Mendez-Millan et al. (2011) (ref. ^44^) with some modifications. All samples were analysed in triplicate. Each sample was refluxed for 18 h at 85–88 °C in purified water: MeOH (1/9 v/v) with 6% KOH. On the same day, the sample was filtered and then stored overnight in the refrigerator. Thereafter, water was added and the pH adjusted to pH 2.0 with HCl. Target compounds were then extracted a total of five times with dichloromethane by phase separation, combining the organic phase with the target compounds in a round-bottom flask. To ensure complete removal of remaining water, the sample was passed over Na_2_SO_4_ and dried under N_2_. Before GC analysis, deuterated eicosanoic acid (D_39_C_20_; Cambridge Isotope Laboratories, Inc.) was added to the sample as an internal standard, followed by silylation at 80 °C for 1 h using N,O-Bis-(trimethylsilyl)-acetamid (BSA; Merck) as described in ref. ^45^. Compounds were then quantified using the same GC system as for lignin. The temperature programme was starting at 50 °C and kept isothermal for 4 min, increasing to 150 °C at a rate of 4 °C min^−1^ and at a rate of 3 °C min^−1^ to 320 °C, where temperature was held again for 40 min. Differentiation of compounds specific for woody (suberin) and leaf (cutin) inputs was not possible in a meaningful way. On the one hand, the above-ground grass biomass contained several compounds in high concentrations, which are usually used to characterize woody inputs (C_22_diacid, ωOHC_18_-ωOHC_24_), leaving only two compounds for the characterization of below-ground inputs (C_18_ and C_20_ diacids). On the other hand, there were only two markers specific for leaf inputs, which were additionally of very low concentrations (9 or 10, ωdiOHC_16_ and ωOHC_14_). Thus, only the sum of all hydrolysable lipids is shown.

### Data analysis

All statistical analyses were conducted using R version 4.1.1^46^ on the RStudio interface^47^. Normality, homoscedasticity and model fit were assessed using residual plots, Shapiro–Wilk test and Levene test. Outliers were assessed using box plots, rosnerTest in the EnvStats package and residual plots. The effect of outlier exclusion was tested by comparing model fit with and without outliers. Data were log transformed to improve model fit. The warming effect was tested using mixed-effect models using gls and *l*me functions in the nlme package. Restricted maximum likelihood was used with the random-effect block (*n* = 3) and the fixed-effects treatment, depth and their interaction. As previous results^26^ indicated more-pronounced effects in the subsoil, models were run on a subset of depth increments (below 20 cm), which is specified when we report the results. We used a significance level of *P* < 0.05. Pearson correlation was calculated using cor.test function as a two-tailed correlation on non-transformed individual data points.

## Online content

Any methods, additional references, Nature Portfolio reporting summaries, source data, extended data, supplementary information, acknowledgements, peer review information; details of author contributions and competing interests; and statements of data and code availability are available at 10.1038/s41561-023-01142-1.

## Data Availability

The data used in this study are available from ESS-DIVE repository (10.15485/1915661).

## Code availability

We used no unreported custom computer code or algorithm.
